# Antiseptics

**Published:** 1889-10-19

**Authors:** 


### ANTISEPTICS.

A paper by C. T. Kingzett, F.I.C., F.C.S., "On the Comparative Antiseptic Value of Chlorides, Nitrates, Sulphates, and other Chemical Substances,"† demands atten-

† *Provincial Medical Journal*, August, 1889.

tion. The author remarks that it is now generally taken for granted that micro-organisms do not of themselves give rise to disease, but that under favourable circumstances they lead to the formation of certain chemical products which are poisonous.

After detailing a number of experiments, the author states that one of the most interesting results is the establishment of the fact of the pronounced antiseptic value of chloral; and only somewhat less certain is the antiseptic value of potassium bisulphate. Sulphate of quinine, if administered with sulphuric acid, may be expected to act, not only as a tonic, but also to produce an antiseptic state of the body. But experience, so far, seems to point to corrosive sublimate as the most powerful antiseptic yet discovered; but, unfortunately, the poisonous nature of the substance disqualifies it for general use. It is also deficient in power of oxidation. It is capable of killing germs or spores, but has no power of oxidation over toxic products. The author has therefore introduced a compound solution of this substance with peroxide of hydrogen. In conclusion, the author mentions "Sanitas" fluid as being of great value as a popular disinfectant.

